# The association between *Helicobacter pylori* with nonalcoholic fatty liver disease assessed by controlled attenuation parameter and other metabolic factors

**DOI:** 10.1371/journal.pone.0260994

**Published:** 2021-12-13

**Authors:** Yoo Min Han, Jooyoung Lee, Ji Min Choi, Min-Sun Kwak, Jong In Yang, Su Jin Chung, Jeong Yoon Yim, Goh Eun Chung

**Affiliations:** Division of Gastroenterology and Hepatology, Department of Internal Medicine, Seoul National University Hospital Healthcare System Gangnam Center, Seoul, Korea; Osaka City University Graduate School of Medicine, JAPAN

## Abstract

**Aim:**

Existing studies have suggested an association between *Helicobacter pylori* (*Hp*) infection and nonalcoholic fatty liver disease (NAFLD). We investigated the relationship between *Hp* infection and NAFLD using controlled attenuation parameter (CAP) and other metabolic factors.

**Method:**

We conducted a retrospective cohort study of apparently healthy individuals who underwent liver Fibroscan during health screening tests between January 2018 and December 2018. Diagnosis of *Hp* infection was based on a serum anti-*Hp* IgG antibody test and CAP values were used to diagnose NAFLD.

**Results:**

Among the 1,784 subjects (mean age 55.3 years, 83.1% male), 708 (39.7%) subjects showed positive results of *Hp* serology. In the multivariate analysis, obesity (body mass index ≥25) (odds ratio [OR] 3.44, 95% confidence interval [CI] 2.75–4.29), triglyceride (OR 2.31, 95% CI 1.80–2.97), and the highest tertile of liver stiffness measurement (OR 2.08, 95% CI 1.59–2.71) were found to be associated with NAFLD, defined by CAP ≥248 dB/m, while *Hp*-seropositivity showed no association with NAFLD. Serum levels of HDL cholesterol significantly decreased in subjects with *Hp*-seropositivity compared to *HP-*seronegativity in both groups with and without NAFLD (*P*<0.001).

**Conclusion:**

While *Hp* seropositivity was not associated with CAP-defined NAFLD, serum HDL cholesterol level were negatively associated with *Hp*-seropositivity in both groups with and without NAFLD. Further clinical and experimental studies are necessary to determine the association between *Hp* infection and NAFLD.

## Introduction

Non-alcoholic fatty liver disease (NAFLD) is the most common liver disease with increasing prevalence as 25% globally, and 27% in Asia [1]. NAFLD is closely linked to various metabolic disorders such as obesity, type 2 diabetes, and cardiovascular disease, and has been considered as a hepatic manifestation of metabolic syndrome [2]. Although biopsy has been regarded as the gold standard for diagnosis and quantitation of hepatic steatosis, its use in clinical practice is extremely limited due to its invasiveness and possible sampling error, especially in asymptomatic subjects without overt liver disease. Therefore, ultrasonography has been recommended as the first-line modality in the clinical practices [3]. Besides imaging modalities, a controlled attenuation parameter (CAP) during transient elastography using FibroScan® shows high sensitivity for detecting low-grade steatosis and good correlation with grades of steatosis. Thus, CAP measurement constitutes a good non-invasive biomarker of hepatic fat or fatty liver, and it enables early and noninvasive detection of NAFLD at the subclinical stage [4–6].

*Helicobacter pylori (Hp)* is a Gram-negative microorganism that infects more than half of the global population [7]. While *Hp* is considered to play a causative role in many gastrointestinal diseases such as chronic gastritis, peptic ulcers and gastric cancer [8, 9], its role in extra-gastric diseases including metabolic syndrome, hematologic and cardiovascular diseases has also been studied [10]. Although many studies have reported the relationship of *Hp* infection with NAFLD [11], the association between *Hp* infection and NAFLD is still controversial [12].

We evaluated the association between *Hp* infection with NAFLD defined by CAP using FibroScan® which gives an objective value for early diagnosis of hepatic steatosis and fibrosis, and other metabolic parameters in asymptomatic population.

## Methods

### Study population

**Fig 1:**
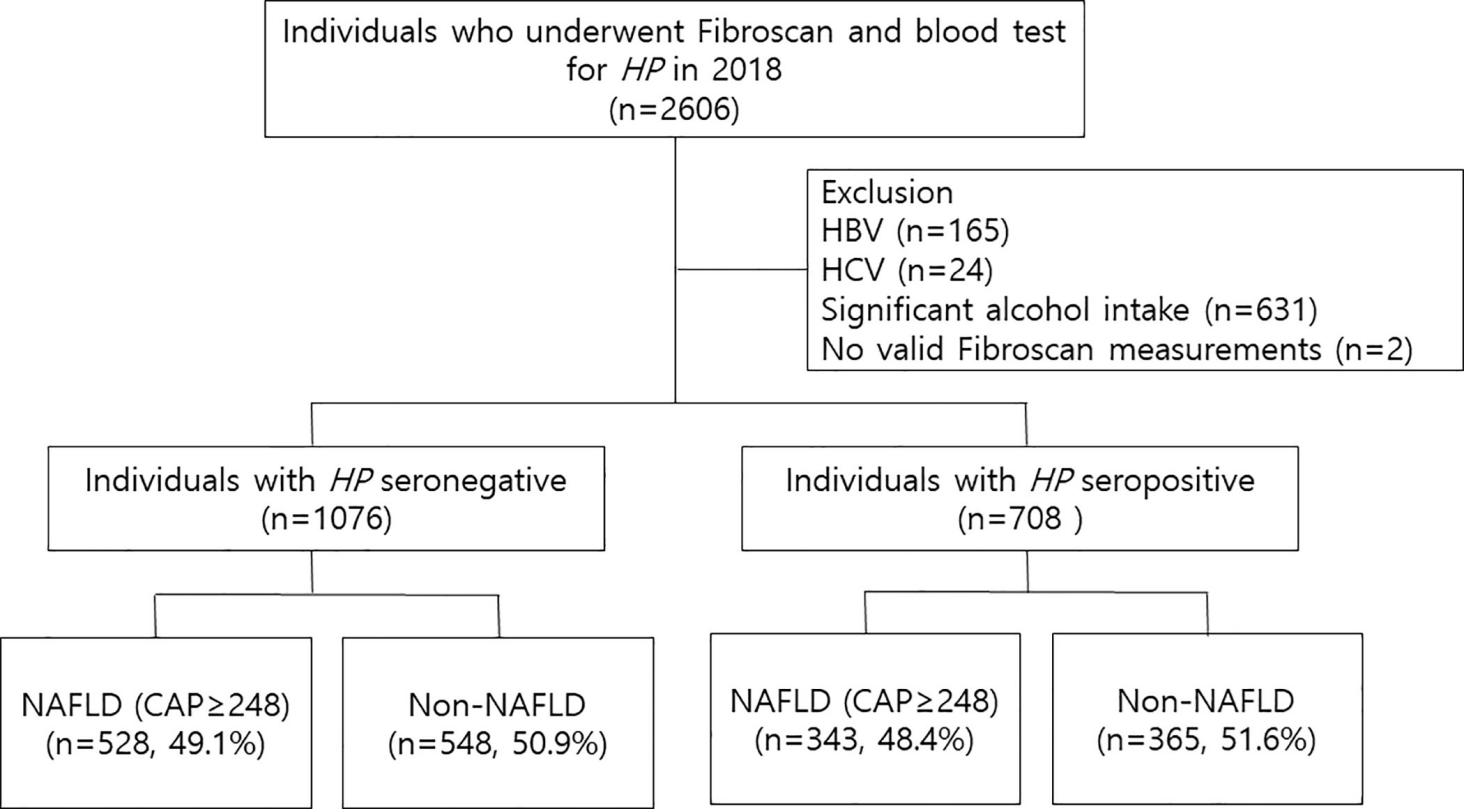
Flow diagram of study population. *HP*, helicobacter pylori; HBV, hepatitis B virus; HCV, hepatitis C virus; NAFLD, non-alcoholic fatty liver disease; CAP, controlled attenuation parameter.

This observational study used a previously conducted retrospective cohort including subjects who underwent routine health check-ups at the Seoul National University Hospital Healthcare System Gangnam Center from Jan 2018 to Dec 2018 [13]. Briefly, individuals either voluntarily underwent examinations or were supported by their employers for health check-ups. They were mostly free of symptoms and underwent tests including FibroScan® (Echosens, Paris, France) and *Hp* serology test on the same day. Initially, a total of 2,606 subjects were enrolled. We excluded 820 subjects who showed any potential cause of chronic liver disease; 165 were positive for the hepatitis B virus, 24 were positive for the hepatitis C virus and 631 had significant alcohol intake (> 210 g/week for males and > 140 g/week for females) [14], and 2 subjects with no valid Fibroscan measurements. Finally, 1,784 subjects were included in the final analysis (Fig 1).

The study protocol followed the guidelines of the Declaration of Helsinki of 1975, as revised in 1983. The protocol was approved by the Institutional Review Board of Seoul National University Hospital (No. 2005-051-1121). Informed consent was waived by the board as researchers accessed and analyzed only de-identified data.

### Measurement of anthropometric and laboratory parameters

The methods employed in this study have been previously described in detail [15]. Data regarding past medical history, comorbidities, and medication history were obtained using subject-recorded questionnaires. The amount of alcohol each patient consumed was calculated. Anthropometric and laboratory parameters were taken on the same day of the health check-ups. Waist circumference was measured in a horizontal plane around the abdomen at the level just above the uppermost lateral border of the iliac crest, just below the lowest rib, and midway between both sites. Body weight (kg) and height (cm) were measured using a digital scale in a standing position. Body mass index (BMI) was calculated by dividing weight (kg) by the squared value of height (m^2^). Blood pressure was measured at least twice after a resting period, and mean values of the measurements were recorded. Hypertension was defined as blood pressure ≥140/90 mmHg or receiving antihypertensive medications. Laboratory tests included serum total cholesterol, triglycerides, high-density lipoprotein (HDL) cholesterol, fasting glucose, and glycated hemoglobin A1c. Diabetes was defined as fasting blood glucose ≥126 mg/dL or glycated hemoglobin A1c ≥ 6.5% or receiving glucose-lowering agents. All blood samples were collected after a 10-hour overnight fast and tests were performed using standard laboratory methods.

### Measurement of *Helicobacter pylori* infection

Diagnosis of *Hp* infection was based on the results of a serum anti-*Hp* IgG antibody test using a commercially available chemiluminescent microparticle immunoassay kit (Immulite ® 2000 CMIA, Siemens, Germany) as described previously [16]. In brief, it is a solid-phase, two step chemiluminescent enzyme immunoassay. The *Hp* IgG in diluted serum sample bound with antigen-coated bead enclosed within a test unit. After removing unbound serum by centrifugation, an alkaline phosphatase-labeled anti-human IgG is introduced. The unbound conjugate was removed by a centrifugation. Then, the chemiluminescent substrate, a phosphate ester of adamantyl dioxetane, is added and underwent hydrolysis in the presence of alkaline phosphatase to yield an unstable intermediate. The emission of light was measured by the luminometer, which was related to the presence of anti-*Hp* IgG in the sample [17]. Values higher than 1.10 IU/mL were considered positive [18]. The *Hp* IgG kit has a sensitivity of 91% and a specificity of 100% [7].

### Measurement of NAFLD using CAP and liver stiffness

CAP and liver stiffness measurements (LSM) were obtained by FibroScan® using an M or XL probe (Echosens, Paris, France) as described previously [19]. Briefly, the procedure was performed by an experienced investigator who was unaware of the patients’ clinical information. The patient lies in a dorsal decubitus position with the right arm fully abducted. Fibroscan® was performed on the right hepatic lobe through the intercostal spaces. The CAP score was expressed as median in dB/m values and the LSM values were expressed as the median kilopascals (kPa). LSM values were considered reliable if 10 valid measurements were obtained and the interquartile range/median of the measurements <0.3 or when the LSM median was <7.1 kPa [20]. All of the patients with 10 valid shots were included in the analysis. In this study, two CAP values of 248 and 268 dB/m were used to define NAFLD [6] and LSM tertile 1 was represented as the lowest values (i.e., T1 ≤ 3.2 kPa, T2; 3.3–4.0 kPa, and T3 ≥ 4.1 kPa).

### Statistical analysis

Continuous variables were expressed as mean ± SD for continuous variables for normally distributed continuous variables, and as median (interquartile range) for non-normally distributed variables. To test for normality, the Kolmogorov-Smirnov test and the normal Q-Q plots were used and log transformations were performed for non-normally distributed variables. Categorical variables were expressed in number and percentage. The comparison of baseline characteristics according to the *Hp* serology was conducted using independent t-tests for continuous variables and the chi-square test for categorical variables. To evaluate the parameters that affect NAFLD, univariate and multivariate logistic regression analyses were performed. Multivariate analyses were adjusted for age, sex, hypertension, diabetes, BMI, fasting glucose, triglyceride, HDL-cholesterol, presence of *Hp* and LSM. All statistical analyses were performed using SPSS 22.0 (SPSS Inc., Chicago, IL, USA), and *P* values <0.05 were considered statistically significant.

## Results

### Clinical characteristics of study population

The mean age of our study population was 55.3 years and 83.1% of the subjects were male. Among the 1,784 subjects, 708 (39.7%) subjects showed positive results of *Hp* serology. Clinical characteristics according to *Hp* serology are summarized in Table 1. Individuals in the *Hp*-seropositive group were older and had lower serum levels of HDL-cholesterol than those in the *Hp*-seronegative group (*P*<0.05). There was no difference in sex composition, serum levels of cholesterol, triglyceride, fasting glucose and hemoglobin A1c, prevalence of hypertension, diabetes, and obesity (BMI ≥25 kg/m^2^) between the two groups. The prevalence of NAFLD defined by CAP≥248 and ≥268 dB/m, and tertiles in LSM values were not different between the two groups. Among the total study population, 50 subjects (2.8%) used XL probe. The mean values of CAP and LSM were higher in subjects using XL probe compared to M probe users (249 vs 326 dB/m and 3.8 vs 4.9 kPa, both *P*<0.05).

**Table 1:** Comparison of baseline characteristics according to Helicobacter pylori serology.

	*Helicobacter pylori* seronegative (N = 1076)	*Helicobacter pylori* seropositive (N = 708)	*P*-value
Age (years)	54.9 ± 10.1	55.9 ± 9.3	0.032
Male, *n* (%)	897 (83.4)	586 (82.8)	0.849
BMI (kg/m^2^)	24.5± 3.2	24.4 ± 3.1	0.469
BMI ≥25 (kg/m^2^)	442 (41.1)	282 (39.9)	0.616
Systolic blood pressure (mmHg)	119.7 ± 14.1	120.4 ± 14.3	0.332
Diastolic blood pressure (mmHg)	78.6 ± 10.4	79.4 ± 10.4	0.175
Diabetes mellitus, n (%)	148 (13.8)	100 (14.1)	0.825
Hypertension, n (%)	237 (22.0)	168 (23.7)	0.401
Total cholesterol (mg/dL)	189.2 ± 36.4	190.2 ± 39.6	0.932
Triglyceride (mg/dL) ⁺	104 (72–147)	104 (75–151)	0.053
HDL-cholesterol (mg/dL)	52.8 ± 13.1	50.3 ± 12.1	<0.001
Fasting glucose (mg/dL)	104.6 ± 21.8	104.7 ± 21.8	0.932
Hemoglobin A1c (%)	5.8 ± 0.8	5.8 ± 0.8	0.968
*Transient elastography*			
Controlled attenuation parameter, dB/m⁺	246 (212–289)	245 (213–282)	0.725
CAP≥248	528 (49.1)	343 (48.4)	0.796
CAP≥268	403 (37.5)	247 (34.9)	0.270
Liver stiffness measurement, kPa⁺	3.6 (3.1–4.3)	3.5 (3.0–4.3)	0.903
Tertile 1^st^ (-3.2)	344 (32.2)	251 (35.5)	0.311
Tertile 2^nd^ (3.3–4.0)	387 (36.0)	243 (34.3)	
Tertile 3^rd^ (4.1-)	345 (32.1)	214 (30.2)	

Data are shown as the mean ± SD.

⁺ median (interquartile range)

BMI, body mass index; CAP, Controlled attenuation parameter; HDL, high-density lipoprotein

### Association between *Helicobacter* infection and NAFLD assessed by CAP

Table 2 shows the association of each parameter with NAFLD defined by CAP≥248 dB/m using univariate logistic regression analysis. Male sex, hypertension, diabetes, BMI, fasting glucose, triglyceride, HDL-cholesterol, and LSM were significantly associated with NAFLD (*P*<0.05). There was no association between *Hp*-seropositivity and NAFLD. Regarding the multivariate analysis, obesity (BMI ≥25 kg/m^2^) (OR 3.44, 95% CI 2.75–4.29), triglyceride (OR 2.31, 95% CI 1.80–2.97), and the highest tertile of LSM value (OR 2.08, 95% CI 1.59–2.71) were found to be associated with NAFLD. *Hp*-seropositivity was not significantly associated with NAFLD. When using different cut-off values of CAP as 268 dB/m for defining NAFLD, similar results were found (S1 Table).

**Table 2:** Factors associated with NAFLD, defined as CAP≥248 dB/m.

	Univariate analysis			Multivariate analysis		
Variables	Odds ratio	95% CI	*P*-value	Odds ratio	95% CI	*P*-value
Age, years	1.00	1.00–1.01	0.404	1.00	0.99–1.01	0.771
Male	1.63	1.26–2.10	<0.001	1.21	0.88–1.65	0.236
Hypertension	1.64	1.31–2.05	<0.001	1.21	0.93–1.56	0.154
Diabetes mellitus	2.15	1.63–2.85	<0.001	1.00	0.63–1.59	0.989
Body mass index, kg/m^2^	1.44	1.38–1.51	<0.001			
BMI ≥ 25, kg/m^2^	4.87	3.96–5.97	<0.001	3.44	2.75–4.29	<0.001
Fasting glucose, mg/dL	1.02	1.01–1.02	<0.001	1.01	1.00–1.02	0.066
Total cholesterol, mg/dL	1.00	1.00–1.00	0.471			
Triglyceride, mg/dL⁺	3.68	3.00–4.53	<0.001	2.31	1.80–2.97	<0.001
HDL cholesterol, mg/dL	0.97	0.96–0.97	<0.001	0.99	0.98–1.00	0.074
Presence of *H*. *pylori*	0.98	0.81–1.18	0.798	0.96	0.78–1.19	0.719
LSM, kPa	1.27	1.16–1.39	<0.001			
LSM, Tertile 1^st^	1 (reference)		<0.001*	1 (reference)		<0.001*
Tertile 2^nd^	1.44	1.15–1.81	<0.001	1.18	0.91–1.51	0.207
Tertile 3^rd^	2.99	2.36–3.80	<0.001	2.08	1.59–2.71	<0.001

CAP, controlled attenuation parameter; CI, confidence interval; HDL, high-density lipoprotein; *H*. *pylori*, helicobacter pylori; LSM, liver stiffness measurement; NAFLD, nonalcoholic fatty liver disease

⁺ Log transformed

*P for trend

Multivariable analyses were adjusted for age, sex, hypertension, diabetes, body mass index, fasting glucose, triglyceride, HDL-cholesterol, presence of *H*. *pylori* and LSM

Since obesity is the most significant etiology of NAFLD, we performed stratified analysis according to obesity to clarify the influence of *Hp* infection on NAFLD. As a result, *Hp*-seropositivity was not significantly associated with NAFLD in both non-obese and obese individuals (Table 3).

**Table 3:** Factors associated with NAFLD, defined as CAP≥248 dB/m according to obesity.

	Non-obese							Obese									
	Univariate analysis			Multivariate analysis⁺			Univariate analysis			Multivariate analysis^§^		
Variables	OR	95% CI	*P*-value	OR	95% CI	*P*-value	OR	95% CI	*P*-value	OR	95% CI	*P*-value
Age, years	1.00	1.00–1.03	0.069	1.01	0.99–1.02	0.527	0.99	0.98–1.01	0.440	1.00	0.98–1.02	0.960
Male	1.32	0.96–1.81	0.086	1.58	1.07–2.35	0.023	0.84	0.47–1.50	0.559	1.40	0.73–2.68	0.313
HTN	1.63	1.19–2.22	0.002	1.18	0.83–1.67	0.350	1.25	0.86–1.81	0.237			
Diabetes	1.71	1.15–2.56	0.009	1.40	0.73–2.71	0.315	1.87	1.19–2.95	0.007	0.92	0.45–1.90	0.818
BMI, kg/m^2^	1.56	1.42–1.71	<0.001	1.54	1.39–1.70	<0.001	1.29	1.17–1.42	<0.001	1.22	1.11–1.36	<0.001
FBG, mg/dL	1.01	1.01–1.02	<0.001	1.00	0.99–1.01	0.660	1.02	1.01–1.03	0.001	1.01	1.00–1.03	0.124
TC, mg/dL	1.00	1.00–1.01	0.032	1.00	1.00–1.01	0.219	1.00	0.99–1.00	0.083			
TG, mg/dL⁺	3.09	2.33–4.08	<0.001	2.31	1.63–3.29	<0.001	2.33	1.65–3.30	<0.001	1.68	1.12–2.53	0.012
HDL-C, mg/dL	0.98	0.98–0.99	<0.001	1.00	0.98–1.01	0.757	0.98	0.98–0.99	0.001	0.99	0.97–1.01	0.233
*H*. *pylori*	0.92	0.71–1.19	0.518	0.89	0.67–1.19	0.431	1.13	0.81–1.58	0.459	1.13	0.79–1.63	0.501
LSM, kPa	1.01	0.96–1.07	0.636				2.06	1.69–2.52	<0.001			
LSM, Tertile 1^st^	1 (ref)		0.247*				1 (ref)		<0.001*	1 (ref)		<0.001*
2^nd^	1.11	0.83–1.49	0.488				1.72	1.15–2.57	0.008	1.15	1.00–2.29	0.053
3^rd^	1.32	0.95–1.83	0.095				5.27	3.40–8.17	<0.001	3.98	2.51–6.30	<0.001

BMI, body mass index; CAP, controlled attenuation parameter; CI, confidence interval; FBG, fasting blood glucose; HDL-C, high-density lipoprotein-cholesterol; *H*.*pylori*, helicobacter pylori; HTN, hypertension; LSM, liver stiffness measurement; NAFLD, nonalcoholic fatty liver disease; OR, odds ratio; TC, total cholesterol; TG, triglyceride

⁺ Log transformed

*P for trend

⁺ Adjusted for age, sex, hypertension, diabetes, body mass index, total cholesterol, fasting glucose, triglyceride, HDL-cholesterol and *H*. *pylori*

^§^ Adjusted for age, sex, diabetes, body mass index, fasting glucose, triglyceride, HDL-cholesterol, *H*. *pylori* and LSM

### Association of metabolic parameters and *Helicobacter* infection

We further investigated the metabolic factors associated with *Hp* infection. When we compared the metabolic parameters including total cholesterol, triglyceride, HDL-cholesterol, fasting glucose and waist circumference according to the presence of *Hp*, serum levels of HDL cholesterol significantly decreased in subjects with *Hp*-seropositivity compared to *Hp-*seronegativity in both groups with and without NAFLD defined by CAP≥248 dB/m (*P*<0.001, Table 4).

**Table 4:** Metabolic parameters and *Helicobacter pylori* infection with or without NAFLD.

Variables	Hepatic steatosis	*H*. *pylori negative*	*H*. *pylori positive*	*P*-value
Total cholesterol, mg/dL	No	189.6 ± 36.1	192.2 ± 39.4	0.222
	Yes	189.8 ± 36.3	190.3 ± 40.3	0.840
Triglyceride, mg/dL⁺	No	93 (64–131)	90 (67–131)	0.305
	Yes	129 (90–177)	131 (92–186)	0.064
HDL cholesterol, mg/dL	No	55.8 ± 13.5	53.2 ± 12.8	<0.001
	Yes	49.9 ± 11.6	48.2 ± 11.5	<0.001
Fasting glucose, mg/dL	No	101.6 ± 20.7	100.6 ± 16.4	0.355
	Yes	108.5 ± 21.4	110.1 ± 25.2	0.262
Waist circumference, cm	No	85.5± 7.5	85.2± 7.7	0.556
	Yes	93.0 ± 8.1	92.7 ± 7.6	0.466

HDL, high-density lipoprotein; *H*.*pylori*, helicobacter pylori; NAFLD, nonalcoholic fatty liver disease

Data are shown as the mean ± SD.

⁺ median (interquartile range)

## Discussion

To the best of our knowledge, our study is the first to investigate an association between CAP as a surrogate marker of liver steatosis and *Hp* infection. In our study, current *Hp* infection was not a risk factor for NAFLD assessed by CAP. Moreover, serum HDL cholesterol level significantly decreased in subjects with *Hp*-seropositivity in both groups with and without NAFLD.

Previous studies have investigated the association between *Hp* infection and NAFLD. Consistent with our results, Baeg *et al*. reported that *Hp* infection was not a risk factor for NAFLD as indicated by hepatic steatosis index or NAFLD liver fat score [21]. Also, no independent association between *Hp* infection and NAFLD was found from a large population study in China [22] and Japan [23]. However, Jiang et al. reported the association between *Hp* infection and NAFLD in the North Chinese population with mildly increased OR 1.22 [24]. Kim *et al*. conducted a large longitudinal study of the Korean population and discovered that *Hp* infection was associated with the development of ultrasonography-diagnosed NAFLD and increased the hazard ratio to 1.21 [25]. A recent meta-analysis reported that *Hp* infection was associated with a higher risk of both prevalent and incident NAFLD (OR 1.20 and HR 1.14, respectively) in middle-aged individual [26, 27]. In most studies, the diagnostic method for NAFLD was ultrasonography while in some cases histology and hepatic steatosis index was used for NAFLD diagnosis. Although ultrasound is a good modality to detect moderate-severe fatty liver, the sensitivity of ultrasound decreases as the hepatic fatty infiltration decreases, so in the presence of liver fat content below 20%, it had a sensitivity of only 55% [3, 28]. In addition, there are limitations of the reliability of such radiologic evaluations including inter- and intra-observer variations [3].

The CAP using FibroScan® has recently been introduced as a noninvasive method to detect and quantify the fat in the liver with adequate sensitivity [29]. A previous study has shown that the CAP values were significantly associated with the severity of hepatic steatosis diagnosed by ultrasonography findings, suggesting that increased CAP could be an early indicator of NAFLD [5]. Moreover, the usefulness of CAP is not limited to the liver. The association between CAP-defined NAFLD and coronary artery plaques [15], increased arterial stiffness [30], and gastroesophageal reflux disease [31] has been reported, suggesting the role of CAP as extra-hepatic manifestations in individuals with NAFLD. When we used CAP for the diagnosis of NAFLD, obesity (BMI ≥25), serum triglyceride and the highest tertile of LSM value were significantly associated with NAFLD consistent with previous results [25], whereas *Hp* seropositivity was not.

As a possible mechanism of linkage between NAFLD and *Hp* infection, *Hp* is thought to contribute in the pathogenesis of NAFLD by increasing insulin resistance and intestinal permeability, and stimulating the release of pro-inflammatory cytokines, shifting the body toward a more lipogenic profile [11, 32]. However, evidence for the association between *Hp* infection and NAFLD remains limited and studies have produced controversial results (Table 5). This may be due to heterogeneity in the study population, method of *Hp* or NAFLD detection among study groups, evaluation of cytotoxin-associated gene A strain [33], and publication bias.

**Table 5:** Summary of relevant studies regarding NAFLD and *Helicobacter pylori* infection.

Reference	Study design	Country	Number of subjects	*Hp* detection	NAFLD detection	Conclusion
Okushin *et al*. [23], 2015	Cross-sectional	Japan	13737	Serum Ig G	Ultrasonography	Negative
Baeg *et al*. [21], 2016	Cross-sectional	Korea	3663	C-UBT	HIS and NAFLD-LFS	Negative
Fan *et al*. [22], 2018	Cross-sectional	China	21456	C-UBT	Ultrasonography	Negative
Kang SJ *et al*. [33], 2018	Cross-sectional	US	5404	Serum Ig G	Ultrasonography	Negative
Jiang et al. [24], 2019	Cross-sectional	China	4081	C-UBT	Ultrasonography	Positive
Kim *et al*. [25], 2017	Longitudinal cohort	Korea	17028	Serum Ig G	Ultrasonography	Positive
Abdel-Razik *et al*. [27], 2018	Longitudinal pilot	Egypt	369	Fecal antigen test	HSI	Positive

UBT: Urea breath test; Ig G: Immunoglobulin G; HSI, hepatic steatosis index; NAFLD-LFS; nonalcoholic fatty liver disease-liver fat score

Since obesity is the most significant etiology of NAFLD, it may reduce the impact of *Hp* infection on NAFLD. When we performed stratified analysis according to obesity, *Hp*-seropositivity was not significantly associated with NAFLD in non-obese population. In several studies, the prevalence of *Hp* seropositivity among bariatric patients was significantly higher compared to the general population control group [34], and active *Hp* infection was associated with histological severity of NAFLD in morbidly obese patients [35], suggesting more close link of *Hp* infection and NAFLD in obese population. Further studies are needed to confirm the role of obesity in the association between *Hp* infection and NAFLD.

Among the metabolic parameters, serum HDL cholesterol level significantly decreased in subjects with *Hp*-seropositivity in both groups with and without NAFLD in this study. Several studies previously reported that *Hp* infection significantly affects serum lipid profiles, consistent with our results [36, 37]. The secretion of inflammatory cytokines by chronic infection of gram-negative bacteria may be related to the change of lipid profiles [38].

## Limitations

Our study has several limitations. First, its cross-sectional design limits the ability to verify causality and we could not infer causal relationships from this study. Second, the diagnosis of NAFLD was not confirmed by biopsy in this study. Although a meta-analysis suggested the optimal cut-off point of CAP for distinguishing normal vs. hepatic steatosis [6], the ideal CAP cut-off values for detecting and grading of steatosis have not yet been established. The differences of the cut off and/or reference value may relate to differences in the study design and populations including disease etiologies, prevalence of obesity and extent of subcutaneous adiposity, and the severity of steatosis, which may influence CAP performances [39–42]. Also, weight reduction may change the CAP values [43]. Third, the serologic test cannot discriminate current and past *Hp* infections. However, the *Hp* infection status was assessed only with serology without using other assessment methods such as urease breath test or a rapid urease test in this study. Due to its cost-effectiveness and non-invasiveness, the serology test is a common method used in the health screening center which is conducted as routine blood sampling. In addition, as various *Hp* antigens are associated differently with metabolic conditions, it would be better to verify multiple *Hp* antigen using multiplex serology [44], however, it was unavailable in this study. Also, we did not have information of *Hp* eradication history. Lastly, this study population of those who underwent health check-ups based on their own initiative may not represent the majority of the general Korean population and this may cause a selection bias.

## Conclusion

While *Hp* seropositivity was not associated with CAP-defined NAFLD, serum HDL cholesterol level were negatively associated with *Hp*-seropositivity in both subjects with and without NAFLD. Further clinical and experimental studies are needed to determine the association between *Hp* seropositivity and NAFLD.

## Supporting information

S1 Table Factors associated with NAFLD, defined as CAP≥268 dB/m. (DOCX)

S1 Appendix The questionnaire for study subjects. (DOCX)

S2 Appendix Dataset. (SAV)

S1 Questionnaire (PPTX)
