# Supplementary material for: The association between Helicobacter pylori with nonalcoholic fatty liver disease assessed by controlled attenuation parameter and other metabolic factors
Source: PLoS One. 2021 Dec 13;16(12):e0260994. doi: 10.1371/journal.pone.0260994 (PMC8668115; doi:10.1371/journal.pone.0260994)
Supplement: S1 Table — (DOCX) [file pone.0260994.s001.docx]

**S1 Table.** Factors associated with NAFLD, defined as CAP≥268 dB/m.

|  | Univariate analysis | | | Multivariate analysis | | |
| --- | --- | --- | --- | --- | --- | --- |
| Variables | Odds ratio | 95% CI | *P*-value | Odds ratio | 95% CI | *P*-value |
| Age, years | 1.00 | 0.99-1.01 | 0.635 | 1.00 | 0.98-1.01 | 0.449 |
| Male | 1.69 | 1.28-2.23 | <0.001 | 1.08 | 0.77-1.52 | 0.646 |
| Hypertension | 1.51 | 1.20-1.89 | <0.001 | 1.12 | 0.86-1.45 | 0.410 |
| Diabetes mellitus | 2.07 | 1.58-2.72 | <0.001 | 1.01 | 0.64-1.60 | 0.959 |
| Body mass index, kg/m^2^ | 1.43 | 1.37-1.50 | <0.001 |  |  |  |
| BMI ≥ 25, kg/m^2^ | 4.87 | 3.96-5.99 | <0.001 | 3.32 | 2.65-4.16 | <0.001 |
| Fasting glucose, mg/dL | 1.02 | 1.01-1.02 | <0.001 | 1.01 | 1.00-1.02 | 0.084 |
| Total cholesterol, mg/dL | 1.00 | 1.00-1.00 | 0.471 |  |  |  |
| Triglyceride, mg/dL⁺ | 3.77 | 3.05-4.70 | <0.001 | 2.44 | 1.88-3.16 | <0.001 |
| HDL cholesterol, mg/dL | 0.97 | 0.96-0.97 | <0.001 | 1.00 | 0.99-1.01 | 0.422 |
| Presence of *H. pylori* | 0.90 | 0.74-1.09 | 0.271 | 0.87 | 0.90-1.10 | 0.241 |
| LSM, kPa | 1.27 | 1.16-1.39 | <0.001 |  |  |  |
| LSM, Tertile 1^st^ | 1 (reference) |  | <0.001* | 1 (reference) |  | <0.001* |
| Tertile 2^nd^ | 1.85 | 1.44-2.38 | <0.001 | 1.50 | 1.14-1.98 | 0.004 |
| Tertile 3^rd^ | 3.97 | 3.08-5.12 | <0.001 | 2.77 | 2.09-3.66 | <0.001 |

CAP, controlled attenuation parameter; CI, confidence interval; HDL, high-density lipoprotein; *H.pylori*, helicobacter pylori; LSM, liver stiffness measurement

⁺ Log transformed, **P* for trend

Multivariable analyses were adjusted for age, sex, hypertension, diabetes, body mass index, fasting glucose, triglyceride, HDL-cholesterol, presence of *H. pylori* and LSM
