# Supplementary figures and images for: The association between Helicobacter pylori with nonalcoholic fatty liver disease assessed by controlled attenuation parameter and other metabolic factors
Source: PLoS One. 2021 Dec 13;16(12):e0260994. doi: 10.1371/journal.pone.0260994 (PMC8668115; doi:10.1371/journal.pone.0260994)

## Slide 1
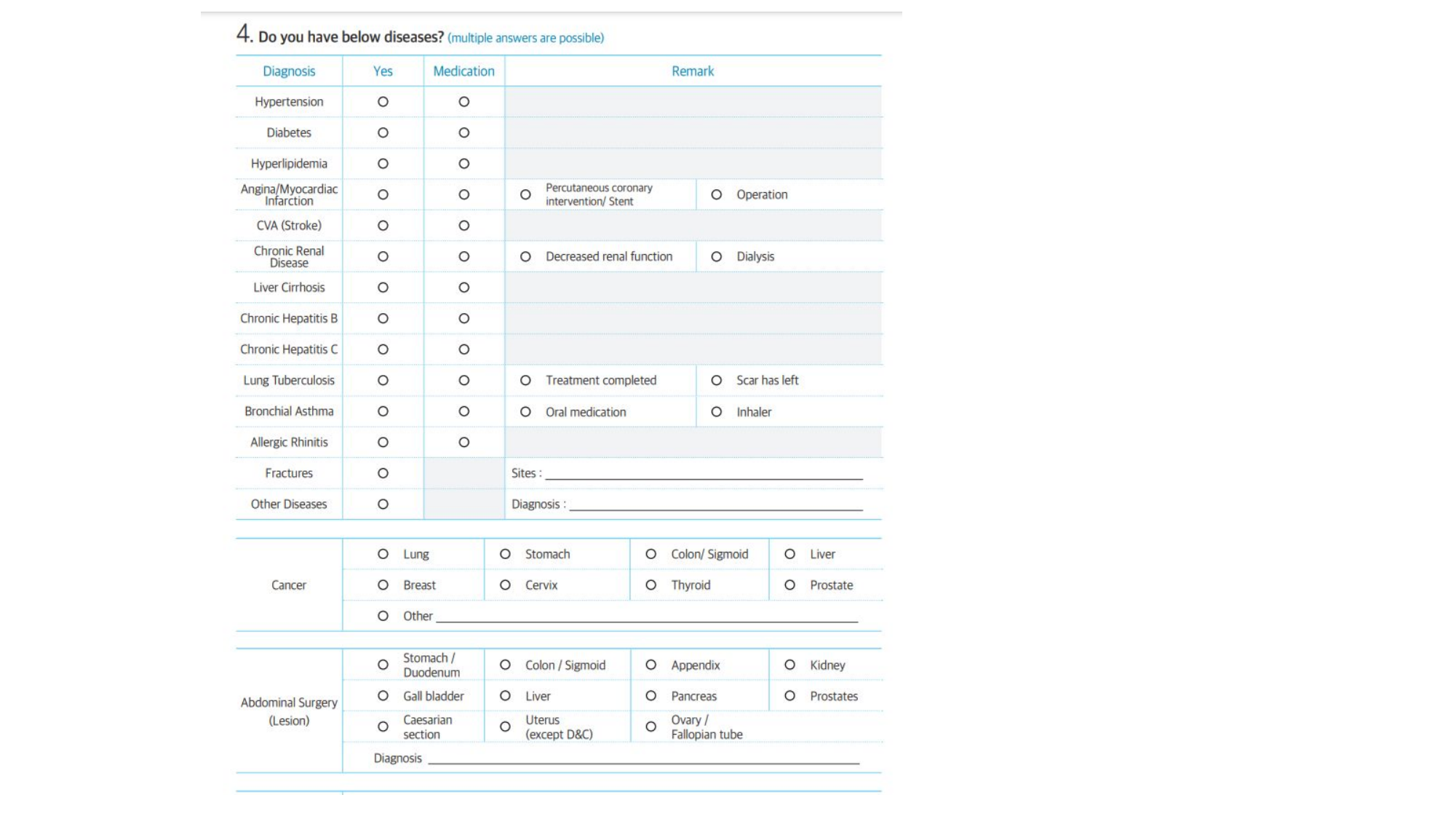

Supplement: S1 Questionnaire — (PPTX) [file pone.0260994.s004.pptx]
